# Supplementary material for: Murine Surf4 is essential for early embryonic development
Source: PLoS One. 2020 Jan 24;15(1):e0227450. doi: 10.1371/journal.pone.0227450 (PMC6980569; doi:10.1371/journal.pone.0227450)
Supplement: Attachment — Submitted filename: 2019_emmer_PLOS_ONE_response_to_reviewers.docx [file pone.0227450.s001.docx]

**Title: Murine *Surf4* is essential for early embryonic development**

We thank the editor and reviewers for their thoughtful evaluation of our initial submission. The decision letter and reviewer comments concisely and accurately described the key findings of our manuscript. The review provided several suggestions which have all been addressed by additional experiments and/or revisions to the text, as outlined in the point-by-point response below. We are grateful for this process, which we believe has resulted in a significantly improved manuscript.

**Reviewer #1:**

**In this manuscript the authors generate a null allele of Surf2, an ER cargo receptor and one they previously identified as interacting with PCSK9 and thus one that may have important roles in cholesterol homeostasis. Overexpression and deletion of Surf2 in cell lines were supportive of this role and they sought to determine if this role was recapitulated in the mouse. They generated 4 lines via a Crispr strategy including 4 putative null lines. Heterozygous animals were generated at expected frequencies but null mice were early embryonic lethal. They find that hets have no changes in plasma levels of cholesterol, PCSK9 or ApoB but do observe significant changes in the abundance of ApoB in the livers of het mice.**

**This paper represents a natural extension of their previous findings and should be of interest to the same audience.**

**Several Major issues include:**

**1) The authors do not present evidence SURF4 is reduced in hets. They do, however repeatedly state that SURF4 (protein) is reduced in heterozygous animals. This information is critical to support the conclusions made herein.**

We have performed further experiments to confirm a reduction in *Surf4* transcripts in heterozygous mice. We both quantified *Surf4* transcripts by qRT-PCR and performed Sanger sequencing of genomic DNA and cDNA, both of which were consistent with a partial reduction of *Surf4* transcripts due to nonsense-mediated decay of the mutant allele (Figure 2). We agree that further confirmation at the protein level would be ideal. However, we have not found an antibody from a commercially available source that specifically recognizes SURF4 when tested on wild-type and SURF4-deficient cell lines. We therefore cannot exclude a compensation at the protein level.

**2) To make definitive conclusions, the heterozygote data presented with a single line should be performed with more than one of the founder lines. The sex and number of animals used in Figs 2 and 3 should also be provided.**

We agree with the reviewer as to the importance of excluding off target effects in gene targeted mice by analyzing multiple founders, where practical. For this reason, we confirmed the loss of the null genotype at weaning in 3 independent mutant lines (Table 2). Gross observation of the colonies also demonstrated no obvious phenotype in the heterozygote offspring from all 3 lines. To limit the mice required and the contribution of genetic drift and nongenetic factors, timed matings and detailed biochemical phenotyping were restricted to littermate controls from a single founder. This point has now been clarified in the Discussion (pages 8-9). The sex and number of animals depicted in Figures 2 and 3 of the original manuscript have now been added to the Figure legends (pages 5 and 6).

**3) No methodology is provide for the data presented in Figure 3. The authors state that liver cell lysates were produced in the text but no information on the antibodies or assays used were provided.**

We thank the reviewers for bringing this to our attention. This information was mistakenly omitted from the original manuscript and is now included in the revised version (pages 11-12).

**It would be important to include a blot demonstrating a reduction in SURF4 in Hets in this figure.**

We agree with the reviewer that this would be a useful confirmation. However, as above, we have not been able to identify a commercial antibody that specifically recognizes murine SURF4 on immunoblotting. To partially address this issue, we have added new data analyzing *Surf4* mRNA in heterozygous mice, demonstrating reduction of the total *Surf4* transcripts in liver with selective loss of the mutant allele, presumably due to nonsense mediated decay. These data are now included in revised Figure 2. However, we cannot exclude compensatory post-transcriptional changes in *Surf4* expression from the wild type allele.

**4) Images of the mutant blastocysts that were created via IVF should be provided in order for experts to decide if there are obvious defects.**

Mutant blastocysts were carefully examined microscopically by an experienced expert (T. Saunders) at the time, with no morphologic differences observed. However, no photographic images were taken at the time. We have revised the Methods and Results (pages 6, 12) to more accurately describe these data.

**Other**

**Abbreviations should be named prior to use: SURF2, ERGIC, LDLR, PCSK9 etc are never defined.**

We have revised the text to include this information.

**Reviewer #2: The manuscript “Murine Surf4 is essential for early embryonic development” by Emmer et al describes the generation of a new mouse knockout model for the anterograde cargo receptor SURF4. The authors used CRISPR/Cas-mediated mutagenesis to target exon2 of the Surf4 gene, generating 4 gene-edited lines (Fig. 1). They then analyzed one of those lines in heterozygous adult mice, by assessing plasma samples for total cholesterol, PCSK9, and ApoB (Fig. 2). PCSK9 and ApoB are two candidate proteins that are predicted to be trafficked by SURF4 from the ER to Golgi based on published literature, and total cholesterol could have been impacted if PCSK9 and ApoB were not synthesized and secreted properly by SURF4-mutant cells. Seeing no effects on Surf4+/- plasma, they analyzed total liver lysates for ApoB, PCSK9, and LDLR (which is regulated by PCSK9) by western blotting (Fig. 3). They found an aberrant accumulation of ApoB in the liver lysates, although this did not affect circulating ApoB levels, as demonstrated by the plasma ELISAs. The authors could not analyze Surf4-/- mice because they were not recovered at weaning from three of their mutant lines (Table 2). They analyzed one of the lines with timed matings to assess when the Surf4-/- embryos died but could not recover live or partially resorbed Surf4-/- embryos at E9.5—the earliest timepoint they analyzed. They also performed in vitro fertilization with Surf4+/- oocytes and sperm and cultured fertilized eggs for 3 days until the blastocyst stage. This approach did generate Surf4-/- blastocysts, so the authors concluded that Surf4-/- embryos died en utero between E3.5 and E9.5 from unknown causes.**

**This manuscript provides the first description of a Surf4-deficient mouse, which is a critical reagent for interpreting the in vitro data that has been generated on SURF4 and its function in different cell types. The authors particularly hoped to validate that the PCSK9 protein—which they had described as being trafficked by SURF4 in vitro (Emmer et al, Elife, 2018)—would also be found to be trafficked in vivo, with possible implications for LDLR surface expression and plasma cholesterol levels. However, the Surf4+/- mice they analyzed did not support a critical role for SURF4 in this capacity. They did, however, find accumulation of ApoB in the livers of Surf4+/- mice, which supports published evidence that SURF4 helps to traffic ApoB for proper secretion from hepatocytes (Saegusa et al, J Cell Biol, 2018). Curiously, this did not alter circulating ApoB plasma levels or cholesterol levels, which the authors could not explain.**

We thank the reviewer for this accurate and concise summary of our findings. As the reviewer points out, despite intracellular accumulation of ApoB in *Surf4*^+/-^ mice, no change in steady state levels of ApoB and cholesterol in plasma is observed. Plasma ApoB levels are determined not only by the rate of secretion but also by clearance, as well as potential downstream effects on complex cholesterol regulatory pathways which could in turn alter the rate of gene expression for ApoB and other related components of this network. In addition, the expected partial reduction in SURF4 expression in heterozygous mice might result in only a modest effect on ApoB secretion. In previous work with another ER cargo receptor (LMAN1), we observed similar hepatic accumulation and unaffected steady state circulating levels for a well-established LMAN1 cargo, alpha-1 antitrypsin, despite homozygous LMAN1 gene disruption (Zhang et al, *Blood* 2011). We have revised the discussion to more fully address these points (page 8).

**While I appreciate the importance of the new Surf4-mutant lines that this manuscript describes, I would have liked to have seen a better analysis of those lines. Specifically, I wish the authors had made some effort to confirm the reduction of SURF4 in their heterozygous adult mice—either by immunoblotting or qPCR. The Surf4-/- blastocysts they generated also could have been analyzed by qPCR. I believe that more careful validation of the mutant lines and of Surf4 reduction is important because Surf4 is known to be subject to alternative splicing (Garson et al, Gene Expr, 1996).**

As note above in our response to point #1 from Reviewer 1, we have confirmed the reduction in *Surf4* mRNA in heterozygous mouse liver, with these data now included in Figure 2. We thank the authors for pointing out the alternative *Surf4* transcripts characterized by Garson et al., which differ only at the 3’ untranslated region, and thus would also be expected to be disrupted by our engineered frameshift mutation in exon 2. The Ensembl genome database confirms this conclusion for these and all known *Surf4* protein coding transcripts. This information has been added to the revised Discussion (page 8).

**In addition, I found the developmental analysis of Surf4-/- embryos to be perfunctory. The fact that no partially resorbed Surf4-/- embryos could be recovered at E9.5 suggests that the embryos die much earlier—potentially around implantation. I found the in vitro generation of Surf4-/- blastocysts to be an interesting approach to assessing survivability of Surf4-/- embryos at early stages of development, although the artificiality of this approach could be misleading in interpreting early Surf4-/- embryonic development. Instead, I would have preferred to see the authors flush blastocysts from pregnant mice at E3.5 to assess the feasibility of early Surf4-/- development. Furthermore, analysis of embryos at peri-implantation (E5.5-6.5) would be a significant improvement to this study, since it would clarify whether Surf4-/- embryos can implant. Methods for dissecting peri-implantation mouse embryos are well described (Shea and Geijsen, JoVE, 2007). Altogether, better analysis of the timepoint at which Surf4-/- embryos die would assist in future studies that will elucidate SURF4 cargo proteins that are critical for early development.**

We agree that a more detailed developmental analysis of the early embryonic lethality observed in *Surf4*^-/-^ mice could be of considerable interest. However, the major focus of our study was the cargo receptor function of SURF4, with the surprising embryonic lethal phenotype indicating the existence of additional cargos and/or functions for SURF4 beyond the limited cargos we and others have previously reported. We are currently generating and characterizing a conditional *Surf4* mutant line with tissue specific Cre recombinases that should greatly enhance these studies, including potential future work by our group and/or others to probe the mechanism of the observed embryonic lethality in germline null mice. However, these studies will obviously take considerable additional time and we hope the reviewers and editor will agree with us that such detailed analysis is beyond the scope of this current manuscript. Nonetheless, we hope that our surprising observation that SURF4 function is required for early embryonic development, and that the secretion of at least some SURF4 cargos may be subtly altered by modest reductions in SURF4 expression, will be of significant interest to a number of other investigators and thus appropriate for publication in PLOSone in its revised form.
